# A New Method of Random Environmental Walking for Assessing Behavioral Preferences for Different Lighting Applications

**DOI:** 10.3389/fpsyg.2017.00345

**Published:** 2017-03-08

**Authors:** Geoffrey R. Patching, Johan Rahm, Märit Jansson, Maria Johansson

**Affiliations:** ^1^Department of Psychology, Lund UniversityLund, Sweden; ^2^Department of Architecture and Built Environment, Lund UniversityLund, Sweden; ^3^Department of Landscape Architecture, Planning and Management, Swedish University of Agricultural SciencesAlnarp, Sweden

**Keywords:** lighting assessment, random walking, structured walking, urban quality, pedestrians

## Abstract

Accurate assessment of people’s preferences for different outdoor lighting applications is increasingly considered important in the development of new urban environments. Here a new method of random environmental walking is proposed to complement current methods of assessing urban lighting applications, such as self-report questionnaires. The procedure involves participants repeatedly walking between different lighting applications by random selection of a lighting application and preferred choice or by random selection of a lighting application alone. In this manner, participants are exposed to all lighting applications of interest more than once and participants’ preferences for the different lighting applications are reflected in the number of times they walk to each lighting application. On the basis of an initial simulation study, to explore the feasibility of this approach, a comprehensive field test was undertaken. The field test included random environmental walking and collection of participants’ subjective ratings of perceived pleasantness (PP), perceived quality, perceived strength, and perceived flicker of four lighting applications. The results indicate that random environmental walking can reveal participants’ preferences for different lighting applications that, in the present study, conformed to participants’ ratings of PP and perceived quality of the lighting applications. As a complement to subjectively stated environmental preferences, random environmental walking has the potential to expose behavioral preferences for different lighting applications.

## Introduction

The role of the built urban environment in supporting people’s health and well-being by facilitating physically active behavior and sustainable travel has received international attention from the World Health Organization (WHO), the United Nations (UN) and the Intergovernmental Panel on Climate Change (IPCC) (de Nazelle et al., 2011). A variety of urban qualities that may enhance public use of urban spaces have been identified; these include large-scale structures but also specific design features, such as smaller-scale elements of urban form; i.e., presence of trees, safe crossings, and adequate lighting (see van Loon and Frank, 2011, for a review). In this regard, a detailed understanding of how such micro-scale urban design qualities lead to improved user experience is called for (Adkins et al., 2012; Harris et al., 2013).

Street lighting is critically important for people’s use of urban spaces, especially for pedestrians at northern latitudes where the number of daylight hours is limited during the winter. However, street lighting generates both environmental and economic costs. The global annual energy used by outdoor lighting is estimated at about 218 TWh (Waide and Tanishima, 2006). Yet, there is potential for saving between 30 and 50% of the total annual lighting energy use (Waide and Tanishima, 2006) by updating existing outdoor lighting installations in terms of design and more energy-efficient light sources (Boyce et al., 2009; Kuhn et al., 2013). New street lighting is associated with large investments and it is important that municipalities choose lighting applications carefully considering energy usage and pedestrian experience (Johansson et al., 2014). Today there is little guidance regarding adequate assessment of pedestrian experience since present standards for road lighting are primarily set from the perspective of motor traffic (Commission Internationale de I’Eclairage [CIE], 2010). This calls for systematic assessments of pedestrian experiences of lighting applications. The present study reports on a behavioral method of assessing pedestrians’ preferences for outdoor lighting applications.

Previous research based on assessments of visual simulations of artificially lit outdoor spaces show that differing lighting applications as well as illuminance levels may fundamentally change the overall impression of public urban environments (Boomsma and Steg, 2014a,b; van Rijswijk, 2015; Nasar and Bokharaei, 2016). Visual simulations of the environment generally provide good representations of the built environment (Stamps, 2015), and may also be sufficient for representing variation in illuminance levels or direction of the light. However, given the complex physics involved, it is difficult to accurately reproduce the quality of the light of each lighting application in simulated environments. In this respect, field studies of pedestrian experiences are required to strengthen the ecological validity of studies employing visual simulations of the built environment alone (e.g., Nasar and Bokharaei, 2016).

In the field, there exists a wide range of instruments designed to capture perceived urban design qualities (Forsyth et al., 2010; Schaefer-McDaniel et al., 2010). Regular environmental scales include self-report measures of users’ perspectives ranging from those capturing general neighborhood qualities (the PREQI; Bonaiuto et al., 2006; Fornara et al., 2010) to those focusing on the streetscape (e.g., the Neighborhood Environment Walkability Scale, NEWS; Saelens et al., 2003), and walking and cycling routes (e.g., the Active Commuting Route Environment Scale, ACRES; Wahlgren et al., 2010). However, these scales do not allow for detailed understanding of pedestrians’ experience of the lit environment. Moreover, it can be difficult to capture perceptions of urban design features in relation to walking without direct exposure to those features (van Cauwenberg et al., 2012). Using ambulatory methods researchers walk with participants in the landscape (Evans and Jones, 2011; Kelly et al., 2011), sometimes using ‘walking probes’ aimed to represent specific sites and to focus the discussion on issues of the built environment (Hein et al., 2008). de Laval (1998) developed ‘walk-through evaluations,’ which is a technique based on a pre-defined route with place-specific stops (probes) to be assessed in positive and negative terms in writing, which are then supplemented by group discussion. Based on this technique Johansson et al. (2016) developed a structured walk that has also been employed to assess pedestrians’ experience of outdoor lighting applications (Rahm and Johansson, submitted). This method has been combined with self-reports of Perceived Outdoor Lighting Quality scale (POLQ, Johansson et al., 2014) covering the experience of strength quality and comfort quality of the outdoor light.

In assessment of outdoor lighting applications, lighting interacts with other properties of the landscape, such as the configuration of built features (Nasar and Fisher, 1992; Nasar and Jones, 1997; Blöbaum and Hunecke, 2005), and vegetation (Luymes and Tamminga, 1995; Lindgren and Nilsen, 2012; Jansson et al., 2013). Therefore, preferences for different lighting applications should also be considered in relation to the landscape properties of the site. According to Küller (1991) preference of the visual experience of the built environment can be described in terms of eight dimensions. In particular, the overarching dimension identified by Küller (1991) is perceived pleasantness (PP), covering the PP, beauty and security of the environment. After Küller (1991) PP is assessed by way of a self-report instrument based on semantic differentials; termed, Semantic Environmental Description (SED). In terms of PP, the SED aims to capture how lighting interacts with other properties of the landscape, and so is incorporated in the present study.

Structured walks and self-report scales, such as the POLQ (Johansson et al., 2014) and SED (Küller, 1991), have many advantages such as ease of administration. However, people’s ratings of the environment are typically based on a single exposure to the environment (de Laval, 1998; Evans and Jones, 2011; Kelly et al., 2011; Johansson et al., 2016). Moreover, self-report questionnaires often rely on paper and pencil format that can be difficult to complete outdoors at night when it is dark (Johansson et al., 2014). Another drawback is that scale items may be interpreted differently by different individuals (see Annett, 2002), which may be exacerbated for people who only have a basic understanding of the native language in which the scale items of the questionnaires are written. In Sweden, time and resource limitations rarely permit translation of scale items into the native languages of all participants, yet it is desirable to recruit a broad range of participants from different backgrounds without language test. An aim of the present study was to develop a new behavioral method of assessing participants’ preferences for outdoor lighting applications, by which to complement existing self-report scales.

As an alternative to self-report scales the method of rank order (Thurstone, 1931) avoids problems associated with subjective interpretation of scale items. Using the method of rank order the lighting applications of interest may be alphabetically labeled and participants merely requested to write down their ranking of the lightings applications in order of preference (see Rajamanickam, 2002). Alone, the method of rank order provides no information about why a participant prefers one lighting application over another, but this method may be used in conjunction with established self-report scales or participants may be asked to give a reason behind their ranking of each lighting application (Boomsma and Steg, 2014b).

A related procedure is the method of paired comparison (see Guilford, 1954; Englund and Hellström, 2012a). The method of paired comparison reduces the process of rank ordering lighting applications to a series of simple judgments of one lighting application against another. Using the method of paired comparison, the lighting applications of interest are factorially combined in pairs. With four lighting applications there are 12 possible combinations [*n*(*n*- 1)] with counterbalanced order or half that number if counterbalanced order is disregarded (cf. Englund and Hellström, 2012a,b, 2013; Patching et al., 2012). The paired lighting applications are presented to a participant one pair at a time in pseudo-random order. In the simplest situation, the participant is requested to choose one of the two paired lighting applications on the basis of whether it is preferred as compared to the other. In the field, this may be achieved by labeling each lighting application alphabetically (e.g., A, B, C, D), and presenting each pair (say A – B) to the participant separately on pre-printed cards. For each pair of lighting applications, responses may be recorded by way of the participant writing down the letter of their preferred lighting application, or by making a tally mark in a paired comparison matrix of lighting application labels (after Hay, 1958).

The method of rank order and related method of paired comparison have a long history in psychology (see Guilford, 1954), and have been used previously to assess the perceived safety of different outdoor lighting conditions (Haans and de Kort, 2012), and acceptability of reduced lighting (Boomsma and Steg, 2014b), to name just two applications in environmental research. Both the method of rank order and method of paired comparison overcome problems associated with interpretation of the scale items of self-report scales, and both methods overcome problems of completing detailed questionnaires at night after dark. However, an important challenge in the field concerns direct exposure of all participants to each lighting application under investigation (Evans and Jones, 2011; Kelly et al., 2011; van Cauwenberg et al., 2012; Johansson et al., 2016), especially when all lighting applications are not visible from a single location in the locale. One possible solution to this problem is to use the method of paired comparison in conjunction with structured walking (after Johansson et al., 2016), whereby each participant is guided to the initial lighting application of the pair and then to the second lighting application of the pair. However, on the basis that each participant is presented with the paired comparisons in different pseudo-random order (see Guilford, 1954, for discussion) the task of guiding each participant to each of the paired lighting applications would have to be done on an individual basis. With a reasonable number of participants (>70), individually guiding each participant to each of the paired lighting applications under comparison would make the comparison task extremely time-consuming and tiring for the study administrator with a task that participants often complain is laborious (Rounds et al., 1978). A further drawback of structured walking is that this method has no potential to reveal how participants’ behaviorally choose to use the lit environment. Yet, anecdotally and evidentially (Larsen and Harlan, 2006), questions remain about mismatches between people’s stated environmental preferences and how the same people actually use their environment. For instance, when questioning colleagues about where they prefer to eat lunch most say that they prefer the stylish and affordable restaurant close to the department, but daily observation of their behavior reveals that most colleagues tend to eat a simple lunch in their office. Consequently, it is not only important to examine participants’ ratings of different lighting applications, but also how participants actually choose to use the environment behaviorally.

One method of examining how participants use the lit environment has been to use eye-tracking equipment with the objective of capturing features critical for pedestrians’ orientation after dark (Davoudian and Raynham, 2012; Luo et al., 2013; Fotios et al., 2015a,b,c). These studies have shown that pedestrians tend to scan the path in front of them and other pedestrians, but say little about how the pedestrians’ experienced the lit environment, because no evaluation of the different lighting applications was undertaken. An alternative method of determining how people use the lit environment is to discretely film and analyze people’s behavior in the environment of interest (Robson, 2011). However, the filming and subsequent analysis of people’s behavior in public places raises a number of ethical concerns (Marx, 1998), which may limit the use of such technology. Indeed, the few existing environmental studies of walking behavior are limited to assessment of pedestrian flow (Herbert and Davidson, 1994; Painter, 1996).

Random environmental walking was conceived as a behavioral complement to structured walks and self-report questionnaires. An advantage of random environmental walking, as compared to self-report questionnaires, is that the random walk procedure proposed may expose participants’ behavioral preferences for different lighting applications. Essentially, the task involves participants repeatedly walking between different lighting applications by random selection of a lighting application and preferred choice or by random selection of a lighting application alone. More specifically, participants are requested to randomly select a lighting application and, by preferred choice, walk actively to that lighting application or make another random selection and walk to that lighting application – for each participant the less favored the lighting application on first random selection the greater the probability of selecting and walking to a more favored lighting application on second random selection. Unlike other procedures such as self-report scales, method of rank order, and method of paired comparison, the random walk procedure proposed involves a physically active behavioral choice that closely resembles the act of walking in an urban environment. Consequently, the procedure provides for the possibility of capturing participants’ behavioral preferences for different lighting applications, which may differ from the same participants’ passively stated preferences.

The random walking procedure described was inspired by the traveling politician problem as detailed by Kruschke (2015, pp. 146–149). The basic idea behind the procedure is random selection of a lighting application followed by a choice (preferred) decision or random selection of a lighting application alone. If the procedure is followed it ensures that participants walk to all lighting applications under investigation but, in line with participants’ preferences for the different lighting applications, participants walk more to their preferred lighting applications. Specifically, the procedure is as follows. First, choose a number of matched urban lighting applications for testing and number the lighting applications accordingly; the number of lighting applications may be any number greater than 2 but the more lighting applications the longer the procedure will take.

With four lighting applications a four-sided, tetrahedral, die can be used to select randomly a lighting application between 1 and 4 (although any device capable of producing discrete random numbers is acceptable; for instance, a mobile phone application). Participants are requested to follow the procedure as detailed below.

Step 1. Throw the die.
Step 2. Walk to the lighting application with the same number as indicated by the die.
Step 3. Throw the die again.

(A) If you prefer the lighting application indicated by the die as compared to your current lighting application walk to the lighting application indicated by the die (if the lighting application indicated by the die is your current lighting application you can choose to stay at that location and repeat Step 3).
(B) Alternatively, you can choose to throw the die again and walk to the lighting application indicated by the die (if the lighting application indicated by the die is the same as your current location stay at that location and repeat step 3).

Repeat Step 3, say 40 times, each time noting the lighting application you walk to. In this case, the precise number of times Step 3 is repeated depends on the accuracy of the results required and on how many participants take part in the study. Note: if a participant has not previously been exposed to the lighting applications under investigation the first few times Step 3 is completed will be indiscriminate. However, as the procedure is followed the participant will walk to every lighting application, more than once, facilitating a behavioral preference on each repetition of Step 3 for a randomly selected lighting application.

### A Computational Simulation Study

In the first instance, we conducted a computational simulation study to (1) verify that the random walk procedure proposed can successfully recover preferences for four different lighting applications, and (2) determine how many participants to test so as to be reasonably (>85%) certain that the random walk procedure captures the overall group’s preferences for four lighting applications. On this basis, four ‘lighting applications’ were computationally defined (#1 to #4) and prior preferences over the four ‘lighting applications’ initially specified in terms of a uniform probability distribution, (#1 = 0.25, #2 = 0.25, #3 = 0.25, #4 = 0.25). The idea, here, was to mimic the assumption that participants initially have no particular preferences for any of the lighting applications. Then on each repetition of Step 3, of the random walk procedure, preferences for each ‘lighting application’ were randomly sampled 16 times from a weighted distribution of preferences defined for each ‘lighting application.’ This sampling procedure was implemented on the grounds that (1) participants’ preferences for the different lighting applications develop over time, (2) participants compare continuously the different lighting applications during the procedure, and (3) a rational choice is made on Step 3 of the procedure. Moreover, we assumed reasonable agreement among participants about the relative rank order of preferences for the different lighting applications, although the precise extent to which each participant prefers each lighting application was assumed to differ between participants. Computationally, this was achieved by defining a unique weighted distribution of ‘lighting application’ preferences for each computationally simulated ‘participant’ by randomly sampling positive numbers from a normal distribution of ‘lighting application’ preferences defined for each ‘lighting application.’ Each sample of ‘lighting application’ preferences for each simulated ‘participant’ was then divided by their sum to form an individual probability distribution of ‘lighting application’ preferences for each simulated ‘participant.’

**FIGURE 1:**
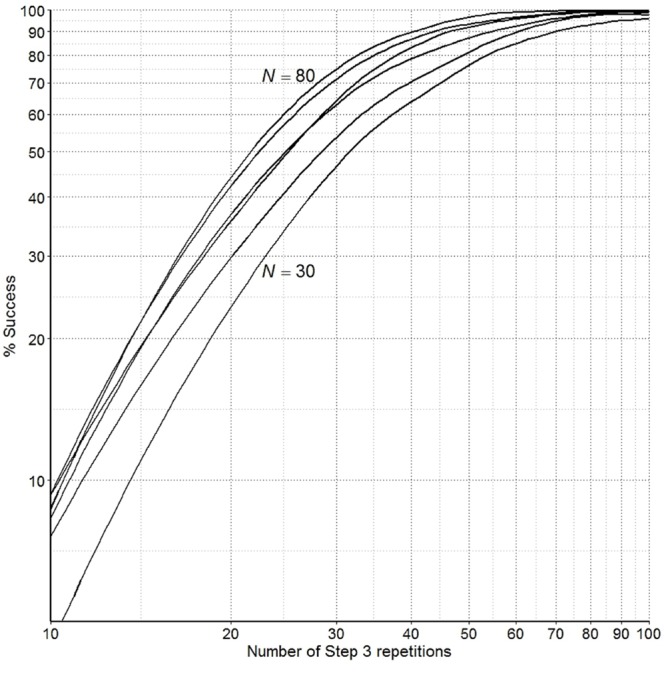
**Number of times out of 100 (% Success) the simulated random environmental walk precisely reproduced the rank order of the simulated ‘participant’s’ preferences for 4 different simulated ‘lighting applications’ for group sizes *N* = 30 to *N* = 80 in increments of 10, for a given number of Step 3 repetitions from 10 to 100 in increments of 2**.

To represent variance among simulated ‘participants’ about their relative preferences for the different ‘lighting applications’ the standard deviation of each sampling distribution of preferences for each ‘lighting application’ was set to 1. The mean of each of these sampling distributions was then determined so that a proportion of the variance defined for each distribution overlapped with the higher or lower ranked ‘lighting application.’ This was done to represent disagreement among participants about the relative ranking of the lighting applications. Conversely, the defined variance unique to each sampling distribution of preferences for each ‘lighting application’ was taken to represent agreement among participants about the relative ranking of the lighting applications. The means of the sampling distributions used for the current simulation were #1 = 100, #2 = 105.84, #3 = 109.76, #4 = 103.28. The unique, non-overlapping, variance defined for each sampling distribution of preferences was taken to represent 95% agreement that ‘lighting application’ #3 is preferred over #2, 80% agreement that #2 is preferred over #4 and 90% agreement that #4 is preferred over #1. So, the overall ‘group’ ranking of preferences for the four ‘lighting applications,’ from most to least preferred, was computationally specified as #3, #2, #4, #1. To recover the simulated preferences for the four ‘lighting applications’ defined, using the random walk procedure proposed, the number of times Step 3 was repeated was increased from 10 to 100 repetitions in increments of two repetitions, and for each number of Step 3 repetitions the random walk procedure was simulated 100 times. **Figure 1** shows the number of times out of 100 (% Success) the simulated random walk precisely reproduced the rank ordering of preferences for the four ‘lighting applications’ as defined over the ‘group,’ for ‘group’ sizes of 30–80 in increments of 10. The indication is that with 80 participants repeating Step 3 40 times each the random walk procedure recovers the precise overall group rank ordering of light application preferences 90% of the time (±5%).^1^

### A Field Test of Random Environmental Walking

A field test was conducted to examine real human participants’ assessment of different lighting applications in a municipal park in Malmö, Sweden. The objective was to determine participants’ preferences for four different lighting applications using the random walk procedure described, and relate the results obtained by random walking to self-report measures completed during a guided structured walk.

## Material and Methods

### Participants

Eighty participants took part in the study – 51 women aged between 20 and 76 years (mean = 44 years), and 29 men aged between 21 and 76 years (mean = 42 years).^2^ All participants were recruited by local advertisement and received 400 SEK for taking part in the field test, and for taking part in another unrelated study that is not reported in the present paper. None of the participants reported any uncorrected visual problems.

### Setting and Lighting Applications

Four lighting applications tenable for use in the City of Malmö were selected by Malmö Streets and Park Department and installed in a small formal garden (area = 500 m^2^), placed in a larger urban park of 45 hectares in total. The choice of setting was made by Malmö Streets and Park Department. For our purpose, the spacing between the four lighting applications was about equal and all lighting applications were within short walking distance of each other (mean distance = 20.5 m).

The garden is rhombic, based on paths of mixed materials (gravel and bricks, along with setts of granite and concrete) and plantations with a mixture of formally cut and free growing plants, surrounded by wooden fences and openings to lawns. The garden design is based on contrasts, both between the surrounding park with voluminous trees (mainly beech – *Fagus sylvatica*) and the more small scale garden, and also inside the garden itself between strict and softer shapes. The garden primarily consists of a system of geometric paths and squared parterres bordered with vegetation in the form of cut hedges of yew (*Taxus baccata*), cut shapes of boxwood (*Buxus sempervirens*) and common ivy (*Hedera helix*). Inside the small squared parterres, there is a varied content with mainly softer shapes, such as free growing plant material, both perennials and small trees, large natural stones, and bird baths. The four lampposts with the lighting applications are all placed along the path which follows the inside of the borders of the rhombic garden. The garden character and landscape properties vary slightly along the path with the lighting applications, as described below.

#### Lighting Application 1

The first lighting application [clear Ceramic Metal Halide (CMH), correlated color temperature (CCT): 2832, color rendering index (CRI): 89, scotopic/photopic-ratio (S/P): 1.27] is located by the entrance of the garden, next to a wooden fence concealing a waste bin. Beside the lighting application there is an open platform with gravel and concrete/granite setts marking the entrance to the garden. In front of the lighting application there is a path of gravel bordered by granite setts. There was no vegetation close to the lamp.

#### Lighting Application 2

From the entrance, the second lighting application [frosted CMH, CCT: 2981, CRI: 82, S/P: 1.29] is further inside the garden than the first lighting application, positioned by a blunt corner of the rhomb. In the surrounding park there are large deciduous trees (beeches – *Fagus sylvatica*). Next to the lighting application, forming a homogenous fond, there is a wooden fence, yew cut as a high ‘hedge end,’ and climbers (Henry’s honeysuckle – *Lonicera henryi*). On the ground there is common ivy (*Hedera helix*). In front of the lighting application there is a 3 m wide path of gravel, and on the other side cut hedges of yew (*Taxus baccata*), forming a corridor by the lamp.

#### Lighting Application 3

The third application [Light-Emitting Diode (LED), CCT: 3912, CRI: 81, S/P: 1.56] is furthest back in the garden positioned at a pointed corner of the rhomb. In the background, there are larger deciduous trees (beeches – *Fagus sylvatica*), a small lawn with cut boxwood balls (*Buxus sempervirens*) and large poles with climbing hop (*Humulus lupulus*). Next to the lighting application there are both low cut hedges, a high ‘hedge end’ of cut yew (*Taxus baccata*), some low free growing lavender (*Lavandula* sp.), and boxwood (*Buxus sempervirens*). On the ground, paving of gravel meets bricks. The brick path widens to one side and on the other side of the path the hedges are turned with the ends toward the lamp, which open up toward the parterres.

#### Lighting Application 4

**FIGURE 2:**
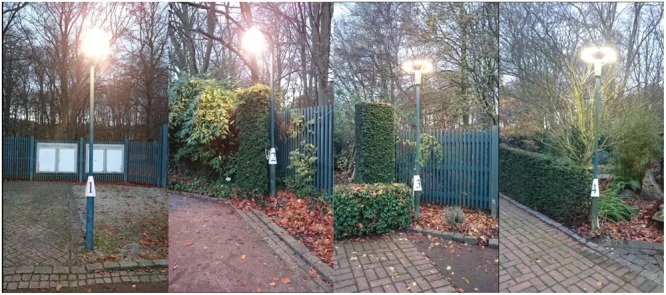
**Photographs of the four lighting applications as detailed in the text, numbered 1–4 from left to right**.

**FIGURE 3:**
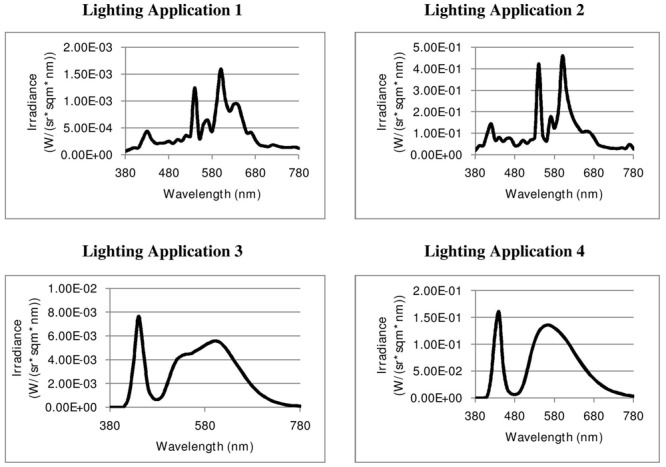
**Spectral power distributions of the four lighting applications under investigation**.

The fourth application [LED, CCT: 4051, CRI: 64, S/P: 1.37] is placed on the outside of a blunt corner at the border of a parterre by a hedge (*Taxus baccata*). On one side the lighting application is positioned inside the branches of a small wedding cake tree (*Cornus controversa*). On the other side of the application are ferns, large nature stones and large ornamental grass. The paving in front of the lamp is brick along with a mixture of concrete and granite setts. On the other side of the path there are cut boxwood balls (*Buxus sempervirens*) and large poles with climbing hops (*Humulus lupulus*) which mark the border to other lawns with larger trees. Lighting Application 4 is positioned in front of a more open setting than Lighting Applications 2 and 3. The 4 lighting applications are shown pictorially in **Figure 2** and their spectral power distributions are shown in **Figure 3**.

In the present setting, it was not possible to view all lighting applications from any one single location within the park. Lighting Applications 2 and 4 were viewable from Lighting Application 1. Lighting Applications 1 and 3 were viewable from Lighting Application 2, and Lighting Application 2 was viewable from Lighting Application 3.

## Measures

Spectral irradiance for each light source was measured with an Avaspec 2048 (Avantes BV). From measurements of spectral irradiance, measures of CCT and CRI were calculated using the software program AvaSoft 7.4 (Avantes BV).

Perceived Outdoor Lighting Quality was assessed using 10, seven-point, rating scales as developed by Johansson et al. (2014). For each lighting application, five items of the POLQ scale assessed Perceived Comfort Quality (PCQ, Cronbach’s alphas = 0.77 – 0.81) and five items assessed Perceived Strength Quality (PSQ, Cronbach’s alphas = 0.82 – 0.85). Participants were also asked to rate Perceived Flicker (PF), on a seven-point rating scale. In addition, PP of the visual environment was assessed using an eight items semantic differential scale (Cronbach’s alpha = 0.71) from the SED as developed by Küller (1991).

For the random environmental walk a tetrahedral die secured in a clear plastic pot was used by each participant to select randomly a lighting application on each repetition of Step 3 of the procedure, as described in the introduction of the present paper. A paper form was provided for each participant to write down the number of the lighting application they walked to on each repetition of Step 3. The POLQ scale, the SED, and form for the random walk procedure, along with instructions about how to complete each part of the study were stapled together and presented to each participant on a clipboard for completion during the study.

### Procedure

All participants undertook the study in small groups of 5–8 participants. Participants were first shown around the site by the study administrator, without requiring them to complete any task. Then, in accordance with the structured walk approach each participant was guided round the four light applications, in serial order #1, #2, #3, #4. All participants were instructed to complete the POLQ scale and the SED, once under each of the four lighting applications. Forty-one participants completed the random walk procedure before completing the POLQ scales and SED. The remaining 39 participants completed the POLQ scales and SED before undertaking the random walk procedure. On each repetition of Step 3, of the random walk procedure, the choice of whether to accept the first random selection and walk to that lighting application or whether to throw the die again and walk to the lighting application selected was made at the lighting application where the participant was standing at the start of Step 3. Instructions about how to complete each part of the study were explained to participants verbally, and the random walk procedure demonstrated to participants behaviorally, immediately prior to participants undertaking each measure. The data were collected during 6 evenings, between 18.00 and 21.00 h when it was dark, between the 11th of November and 1st of December, 2015 (in southern Sweden the sun sets at about 15:30 hrs and no later than 16:00 h during November). The temperature varied between 3 and 11 degrees Celsius (mean = 8.4°C). On 4 evenings it was cloudy, and on 2 evenings it was raining. Participants took, on average, 40 min to complete the study.

This study was carried out in accordance with the rules and regulations laid down by the Ethics Committee for the Swedish Research Council. All participants gave written informed consent in accordance with the Declaration of Helsinki.

### Data Analysis

Data analysis was conducted in three parts. First, linear mixed effects modeling was used to examine the effect of the individual lighting applications on the ratings of PP, PCQ, and PSQ, separately for each subjective measure. Lighting applications 1 – 4 (dummy coded) were entered as fixed effects, and participants and the scale items were entered with their own intercepts as well as by-participant and by-item random slopes for the effect of lighting application. Visual inspection of residual plots did not reveal any obvious deviations of homoscedasticity or normality. To assess the overall fit of each model, *p*-values were obtained by likelihood ratio tests of each model with the lighting application effect against the same model without the lighting application effect (i.e., intercept only models). Graphical inspection of the PF ratings revealed very little difference between the different lighting applications and so PF was not analyzed further.

Second, linear mixed effects modeling was used to examine the behavioral results obtained following the random walk procedure. Lighting applications 1–4 were entered as fixed effects and as random effects participants were entered were with their own intercepts. Residual plots showed no obvious deviations of homoscedasticity or normality. Overall model fit was assessed by a likelihood ratio test, against the same model without the lighting application effect.

Third, relations between the behavioral results obtained following the random walk procedure and participants’ subjective ratings of PP, PCQ, and PSQ, were examined by regression of PP, PCQ, and PSQ, separately on the number of times each participant walked to each lighting application following the random walk procedure. Participants’ ratings of PP, PCQ, and PSQ, were entered as fixed effects and as random effects participants were entered with their own intercept. Again, no obvious deviations of homoscedasticity or normality were found and all model fits were evaluated by likelihood ratio tests against equivalent intercept only models.

All analyses were conducted using R (R Core Team, 2015). The package *lme4 (*Bates et al., 2015) was used for linear mixed effects modeling. No statistically significant effects of gender (male, female), or age were found (all *p*s > 0.05), and so these variables are not included in any of the linear mixed effects models reported in the present paper. Likewise, participants’ ratings of PP, PCQ, PSQ, and the results obtained using the random walk procedure, failed to show any statistically significant differences depending on whether participants completed the self-report scales before or after the random walk procedure (all *p*s > 0.05), and so this variable is not included in the mixed effects models reported.

## Results

### Structured Walks

**FIGURE 4:**
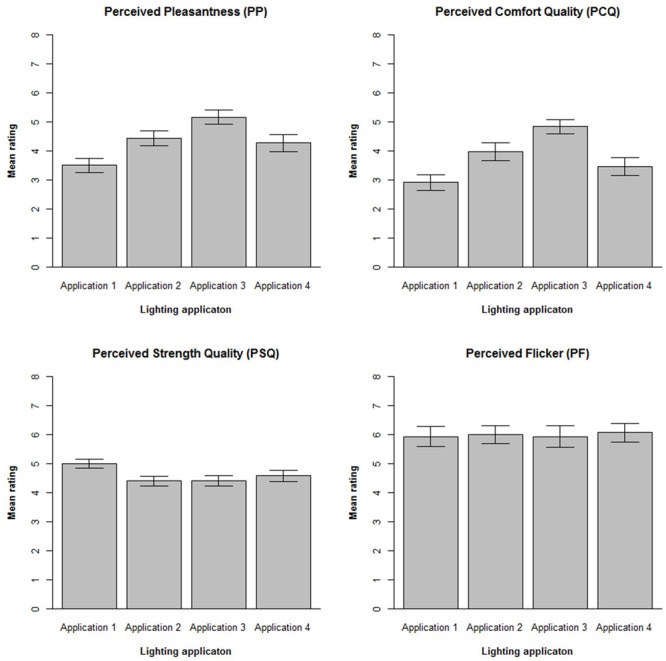
**Mean ratings of the four different lighting applications.** Error bars show 95% confidence intervals calculated using appropriate *t* scores.

Perceived pleasantness was first computed by averaging over the eight items of the semantic differential scale, separately for each of the 4 lighting applications. Likewise, perceived comfort quality and perceived strength quality were computed by averaging over their 5 respective items of the POLQ scale. Participants who failed to complete an item on a respective scale were removed from this analysis, resulting in *N* = 69 for PP, and *N* = 74 for PCQ, and PSQ. Mean averages of the subjective scales (PP, PCQ, PSQ, and PF) over the four lighting applications are shown in **Figure 4**.

Lighting application had a statistically significant effect on ratings of PP, χ^2^(3) = 25.66, *p* < 0.001. Averaged over items, the overall rank order of PP ratings, from highest to lowest, for the four lighting applications is #3, #2, #4, #1. Seventy-two percent of the participants rated PP higher for Lighting Application 3 as compared to Lighting Application 2, 51% rated PP higher for Lighting Application 2 than Lighting Application 4, and 67% of participants rated PP higher for Lighting Application 4 as compared to Lighting Application 1. In similar vein, lighting application had a statistically significant effect on ratings of PCQ, χ^2^(3) = 13.95.8, *p* < 0.001. Overall, the rank order of PCQ ratings, from highest to lowest, for the four lighting applications is #3, #2, #4, #1. Sixty-six percent of the participants rated PCQ higher for Lighting Application 3 as compared to Lighting Application 2, 61% rated PCQ higher for Lighting Application 2 than Lighting Application 4, and 58% of the participants rated PCQ higher for Lighting Application 4 as compared to Lighting Application 1.

Due to high correlations *r* > 0.93 between the ratings of PSQ for the different lighting applications, inclusion of all four lighting applications in analysis of PSQ resulted in problems associated with multicollinearity. To resolve this problem just two lighting applications were entered into the model: the highest PSQ ranked lighting application #1 and lowest PSQ ranked lighting application #3. Overall, the rank order of PSQ ratings, from highest to lowest, for the four lighting applications is #1, #4, #2, #3. Sixty-one percent of the participants rated PSQ higher for Lighting Application 1 as compared to Lighting Application 4, 51% rated PSQ higher for Lighting Application 4 as compared to Lighting Application 2 and, 49% rated PSQ higher for Lighting Application 1 than Lighting Application 3. Statistical analysis failed to show any statistically significant difference between ratings of PSQ for Lighting Application 1 as compared to Lighting Application 3, χ^2^(1) = 0.89, *p* = 0.35.

### Random Environmental Walking

**FIGURE 5:**
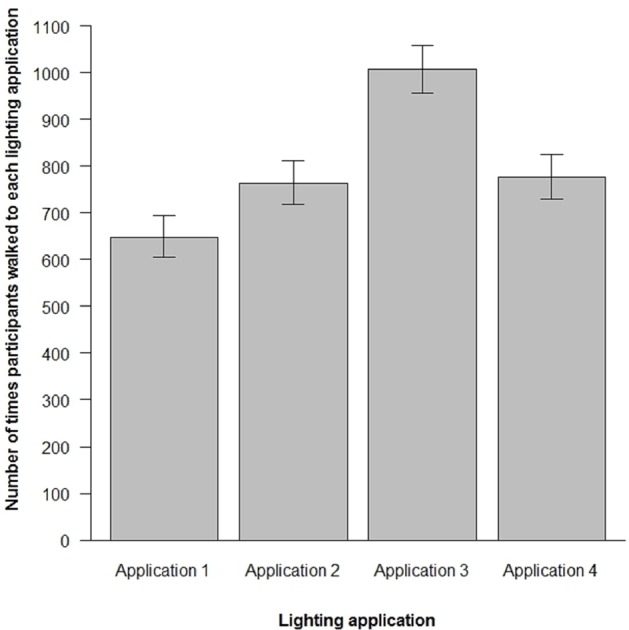
**Overall number of times participants walked to each lighting application following the random walk procedure described.** The error bars represent 95% confidence intervals, calculated following the procedures advocated by Agresti and Coull (1998) for binomial proportions.

All participants successfully completed the random walk procedure noting the number of the lighting application they walked to on each repetition of Step 3 of the procedure. Overall, there were only five repetitions of Step 3 on which four different participants failed to note the number of the lighting application they had walked to. The number of times participants walked to each lighting application following the random walk procedure is shown in **Figure 5**.

Lighting application had a statistically significant effect on the number of times participants walked to each lighting application, χ^2^(3) = 46.62, *p* < 0.001. The overall rank order of the number of times participants walked to each lighting application, from most to least, is #3, #4, #2, #1. Sixty-five percent of the participants walked more to Lighting Application 3 than Lighting Application 4, 48% walked more to Lighting Application 4 than Lighting Application 2, and 56% of the participants walked more to Lighting Application 2 as compared to Lighting Application 1.

Further examination of relations between the results obtained by random walking and the self-report scales show statistically significant relations between the overall number of times participants walked to each lighting application following the random walk procedure and PP, χ^2^(1) = 36.77, *p* < 0.001, and between the overall number of times participants walked to each lighting application and PCQ, χ^2^(1) = 60.49, *p* < 0.001. No statistically significant relations were found between the overall number of times participants walked to each lighting application by random walking and PSQ, χ^2^(1) < 0.001, *p* = 0.99.

## Discussion

Large-scale introduction of energy efficient outdoor lighting applications calls for a broad range of methods by which to systematically assess pedestrians’ preferences for different lighting applications. The current study shows that random environmental walking is a viable technique for use in the field, and in the present case yielded results similar to those obtained by established self-report scales. In this respect, random environmental walking has the potential to become a tool for municipalities to facilitate the choice of outdoor lighting taking into account user perspectives.

The current field test shows reasonable agreement between the results obtained by random environmental walking and the mean ratings of PP and PCQ. PP and PCQ capture the extent to which the light is perceived as soft, natural, warm, mild, and shaded (Johansson et al., 2014). For PP, PCQ, and by random walking, Lighting Application 3 was found to be most preferred and Lighting Application 1 least preferred. In regard to Lighting Applications 2 and 4, mean PP and PCQ ratings were very similar, although a rank ordering of preferences put Lighting Application 2 ahead of Lighting Application 4. In similar vein, the random walk procedure shows that participants walked a similar number of times to Lighting Application 2 as compared to Lighting Application 4. However, in terms of a rank-ordering of the overall number times participants walked to each lighting application, the random walk procedure put Lighting Application 4 over Lighting Application 2.

The difference in the ranking of Lighting Applications 2 and 4 obtained using the random walk procedure as compared to that obtained using the rating scales may due to procedural differences between these two different types of measures. Subjective self-report scales, such as the POLQ scale are useful to determine why participants prefer each lighting application, but fail to provide any information about participants’ behaviorally preferences for the lighting applications. Conversely, random environmental walking potentially provides behavioral information about participants’ preferences for the different lighting application, but does not provide any information about why participants choose to walk more to some lighting applications than others. In this case, it is possible that the more open character around Lighting Application 4 compared to the more narrow position of Lighting Application 2, which may be expected to be preferred for aspects of perceived safety (Jansson et al., 2013), had an influence on the overall number of times participants walked to these lighting applications. In this respect, the present study should be considered as proof-of-concept of the random walk procedure rather than definitive assessment of participants’ behavioral preferences for the lighting sources *per se*. Indeed, without the use of a range of different methods to assess participants’ preferences for different lighting applications, lighting sources installed in urban environments may not necessarily be the lighting applications the majority of people prefer.

A benefit of random environmental walking, as a complement to other methods involving structured walking, is that participants continuously walk around the lit environment of interest in a way that reflects what each participant behaviorally prefers to do in that environment, while ensuring that participants walk to every lighting application. So, the random walk procedure proposed has the potential to reveal how participants behaviorally and repetitively choose to use the lit environment over time, which may not necessarily be the same as participants’ passively stated preferences garnered on single glance. A further benefit of random environmental walking is that the task is not dependent on proficient understanding of the local language.

Self-report rating scales are reasonably easy to administer and are used regularly to assess perceived urban design qualities (Johansson et al., 2014), but as a complement to such scales random environmental walking has the potential to reveal behavioral preferences for different lighting applications that is not reliant on participants’ subjective interpretation of written questions. In the main, the random walk procedure can be demonstrated to participants behaviorally without recourse to opaque language. In this respect random environmental walking is suited for assessment of lighting applications by participants who only have a basic understanding of the native language, and who may have acute difficulty interpreting the nuances of the written language used in the self-report questionnaires. The random walk procedure is linguistically undemanding for participants to complete and may, in this respect, be more inclusive than subjective scales because the procedure facilitates participation of a broader range of user groups. Moreover, random environmental walking may be easily extended to user assessments of indoor lighting applications. Generally speaking, participants reported that they enjoyed the task which many considered to be an amusing game.

On the grounds that each participant followed the random walk procedure as instructed, the simulation study presented in the introduction suggests that with 80 participants taking 40 steps each, we can be more than 85% certain that the random walk procedure captured the overall group’s behavioral preferences for the four lighting applications. However, the simulation study was based on the assumption of greater agreement among participants, about the relative ranking of the lighting applications, than exhibited by the actual participants in the field study. With greater disagreement between participants, than assumed in the simulation study, more steps would be required to precisely capture the group’s behavioral preferences for the four lighting applications. In sum, the more times Step 3 of the random walk procedure is repeated, either by increasing the number of times each individual participant repeats Step 3, or by increasing the overall group size, the greater the certainty that the random walk procedure precisely reveals the behavioral preferences of the participants.

A downside of random environmental walking is that Step 3 of the procedure needs to be repeated a large number of times for accurate assessment of participants’ behavioral preferences for different lighting applications. If in the present study the light sources were changed between sessions and counterbalanced over the four lighting applications it would have been necessary to test at least 320 participants to be reasonably certain of participants’ behavioral preferences for the four lighting applications. As the number of lighting applications to be tested is increased the number of Step 3 repetitions required to capture participants’ behavioral preferences rapidly increases. In this respect, the current random walk procedure proposed is only suitable for application with a limited number of lighting applications (i.e., <6), in a limited number of urban locations. A potentially more efficient method is to diminish the randomness of the procedure, by reducing the random selection of a lighting application on Step 3 to a binary selection between adjacent lighting applications (see Kruschke, 2015, pp. 146–149). This would reduce the number of times Step 3 needs to be completed for accurate assessment of participants’ preferences for different lighting applications, while the behavioral (walking) element of the task could be retained. However, random binary selection of lighting applications would be more difficult to explain to participants, and would make the procedure more like the standard method of paired comparison. In this respect, limiting random selection to a binary selection between adjacent lighting applications may limit the potential of the procedure to capture participants’ behavioral preferences for the different lighting applications. Further investigation is required to examine the effectiveness of reducing the randomness of the procedure to binary selection, as compared to random selection of a lighting application from the total set of lighting applications under investigation.

In conclusion, random environmental walking can reveal participants’ behavioral preferences for different lighting applications that, in the present study, corresponded to participants’ subjective ratings of PP and perceived comfort quality. As compared to subjective rating scales, random environmental walking is a somewhat inefficient procedure but, is less dependent on proficient language skills than self-report scales. As a complement to subjective rating scales of the lit environment, random environmental walking has the potential to provide a new method of assessing pedestrians’ behavioral preferences for different lighting applications.

## Author Contributions

GP and MJo participated in every phase of the study from design to the final manuscript. GP initially conceived of the idea of random environmental walking. JR and MJa contributed significantly to recruitment, data collection, design, and writing.

## Conflict of Interest Statement

The authors declare that the research was conducted in the absence of any commercial or financial relationships that could be construed as a potential conflict of interest. The reviewer EW and the handling Editor declared their shared affiliation, and the handling Editor states that the process nevertheless met the standards of a fair and objective review.

## Footnotes

1 The MATLAB code used to simulate the random walk procedure may be obtained by contacting Geoffrey R. Patching.

2 One man and 7 women did not report their age.
